# Maternal vitamin D and growth of under-five children: a systematic review and meta-analysis of observational and interventional studies

**DOI:** 10.1080/16549716.2022.2102712

**Published:** 2022-08-31

**Authors:** Amare Abera Tareke, Addis Alem, Wondwosen Debebe, Nebiyou Simegnew Bayileyegn, Melese Shenkut Abebe, Hussen Abdu, Taddese Alemu Zerfu

**Affiliations:** a Department of Biomedical Science, College of Medicine and Health Science, Wollo University, Dessie, Ethiopia; b Department of Surgery, Faculty of Medicine, Jimma University, Jimma, Ethiopia; c Global Academy of Agriculture and Food security, Royal (Dick) School of Veterinary Studies, University of Edinburgh (UoE), UK; d College of Medicine and Health Sciences, Dilla University, Dilla, Ethiopia

**Keywords:** Growth, maternal, vitamin D, children, length

## Abstract

**Background:**

Even though previous systematic reviews have reported on the role of prenatal vitamin D on birth outcomes, its effect on child growth is poorly understood.

**Objective:**

To synthesize a systematic summary of the literature on the effect of maternal vitamin D supplementation on the linear growth of under-five children.

**Method:**

This study includes studies (both observational and interventional with a control group) that evaluated the effects of prenatal vitamin D status on child linear growth. The mean child length/length for age with 95% confidence interval (CI) was pooled as the weighted mean difference using a random-effects model. A funnel plot was used to assess potential publication bias.

**Results:**

A total of 45 studies and 66 reports covering a total population of 44,992 (19,683 intervention or high vitamin D group, and 25,309 control or low vitamin D group) were analyzed. Studies spanned from 1977 to 2022. The pooled weighted mean difference was 0.4 cm (95% CI: 0.15–0.65). A subgroup analysis, based on vitamin D supplementation frequency, showed that mothers who supplemented monthly or less frequently had a 0.7 cm (95% CI: 0.2–1.16 cm) longer child. Supplementation with a dose of >2000 international units increased child length at birth. The weighted mean difference was 0.35 cm (95% CI: 0.11–0.58).

**Conclusion:**

The evidence from this review shows that maternal supplementation of vitamin D is associated with increased birth length. This is apparent at higher doses, low frequency (monthly or less frequent), and during the second/third trimester. It appears that vitamin D supplementation during pregnancy is protective of future growth in under-five children. Clinical trials are needed to establish evidence of effectiveness for the frequency and dose of supplementation.

## Background

Vitamin D is a fat-soluble vitamin that increases the absorption of calcium, magnesium, and phosphate. It is used by the body for the development of calcified tissues and helps to prevent rickets [1]. Due to the importance of vitamin D in the human body, its deficiency causing rickets was considered the ‘tip of the iceberg’ [2]. Vitamin D deficiency also causes growth retardation in utero and during childhood, and skeletal deformities that lead to and exacerbate osteopenia, osteoporosis, and increase the risk of fracture [2]. Vitamin D along with calcium plays an important role in the mineralization of bone and has a myriad of other benefits including the prevention of autoimmune diseases, decreased risk of cancer, hypertension, diabetes, and improved immunity [3].

Vitamin D is a steroid hormone; its receptor is located in the nucleus, forming a complex with specific DNA sequences. Vitamin D causes the transcription of a large number of genes, some of which are proteins that promote growth, including growth hormone and insulin-like growth factor-1 [4–7].

There is controversy regarding adequate or optimal levels of serum vitamin D to prevent adverse health consequences. The US Institute of Medicine defined adequate vitamin D in pregnant women as a serum concentration greater than 50 nanomoles per liter (nmol/L) (20 nanograms per milliliter (ng/ml)) [8]. Others argued that the value should be raised to 75 nmol/L (30 ng/ml) [9,10], but the burden remains high. Despite disagreements, inadequate vitamin D is classified as a deficiency at <25 nmol/L [8] and an insufficiency at <50 nmol/L. Adequate vitamin D is generally defined as more than 50 nmol/L [9].

Low vitamin D status varies in populations across the globe. Depending on the Food and Agricultural Organization world regions, the prevalence of serum 25(OH)D < 50 nmol/L ranges from 24% to 49% [10]. Although vitamin D deficiency affects every individual at all levels, diet, supplement use, geographic latitude, cultural and lifestyle factors, and skin pigmentation are important factors. Infants, older individuals, pregnant and lactating women, and individuals having specific disease conditions like cancer are at particular risk of vitamin D deficiency [11,12]. Maternal vitamin D deficiency during pregnancy is also a critical global public health problem, with variations across countries. For example, deficiency in pregnancy has been reported as 81% in Nepal [13], and over 90% in Guizhou, China [14], and Saudi Arabia [15]. A pooled result from a study in African countries reported a prevalence of almost 44% in mothers and newborns [16].

Some countries specify a recommended dietary intake during pregnancy. For example, in the USA, Australia, New Zealand, and Canada, the recommended dietary adequate intake of vitamin D for pregnant women is 200 International Units (IU)/day [17,18]. The UK recommends 400 IU/day during pregnancy [19].

Children less than 5 years old are among the most-affected population segment in terms of vitamin D deficiency. A systematic review and meta-analysis covering countries in the African continent reported the prevalence of vitamin D deficiency at 49% and 25% in newborns and children, respectively, based on a cutoff value of <50 nmol/L [16]. The vitamin D status of infants depends on maternal vitamin D status, the intake of breast milk, and its vitamin content. In India, almost 93% of healthy infants were found to be vitamin D deficient [20].

In the first 6–8 weeks of postnatal life, the vitamin D status of infants is mainly dependent on placental transfer in utero [21]. In most infants, the acquired vitamin D stores are depleted by approximately 8 weeks of age [22]. Thereafter, the infant’s vitamin D supplement is derived from diet, sunlight, and supplementation. Human milk contains an insufficient amount for maintaining optimal vitamin D levels, especially if exposure to sunlight is limited [23]. Exclusively, breastfed infants have hypovitaminosis D due to the poor content of human milk [24,25]. In exclusively breastfed infants, 6 weeks to 6 months postnatal is a critical window for addressing vitamin D deficiency [26].

Since the early 1980s, there have been many vitamin D supplementation trials conducted during pregnancy. However, the interpretation of the results has been complicated by factors such as the type, duration, and dose of supplementation [27]. Systematic reviews have been conducted previously to evaluate the effects of prenatal vitamin D status on the different health outcomes of children. Previous systematic reviews [28–31] investigated the effect of prenatal vitamin D supplementation on birth outcomes. In these studies, the effect of prenatal vitamin D on child growth has remained largely unknown. Despite numerous original studies on maternal vitamin D and child linear growth, comprehensive scientific evidence is lacking. In this review, we ask the question: ‘what effect does maternal vitamin D status have on linear growth in children under the age of five?’ The findings of this synthesis will help inform the scientific community about priority research areas for vitamin D supplementation in child growth.

## Methods

This systematic review and meta-analysis was conducted to synthesize existing evidence on the role of maternal gestational vitamin D supplementation/status in the linear growth of under-five children.

### Search strategy

The search strategy was performed in three stages. In the first stage, relevant Medical Subject Heading (MeSH) and other terms were identified in the literature. In the second phase, full searches were conducted in PubMed, Ovid Embase, and Google Scholar. In the third phase, the bibliographies of relevant studies and university websites were searched to see the presence of eligible studies. The following terms were used to search for relevant articles. The population terms were combined using OR, and the PICO components were combined using AND. MeSH Terms and Asterisk were applied. Population terms were maternal, gestation*, prenatal, antenatal, pregnancy, child, children, under-five, preschool, infant, newborn, and ‘0–59 months’; intervention terms were vitamin D [MeSH Terms], ‘vitamin D’, cholecalciferol, ‘vitamin D3’, ergocalciferol, and alfacalcidol; and outcome was searched using growth disorders [MeSH Terms], ‘linear growth’, stunted, stunting, ‘height for age’, length, ‘length for age’, ‘short stature’, and growth. Filters were used in some databases. This study included studies published from inception to 22 February 2022.

### Study selection

The search included both observational and interventional studies. Interventional/observational studies were required to have a control or comparison group. The outcome (child growth) was extracted as mean length at different age groups or as length for age (LFA)/height for age (HFA) from both interventional and observational studies. Some studies had supplementation in addition to vitamin D (e.g. calcium). We included such studies provided that the intervention and control groups differed only in terms of vitamin D. There was no restriction on when the supplementation/measurement took place, i.e. during the first, second, or third trimester. Childhood growth was evaluated for infants or children under the age of 5 years.

Studies were excluded if the women had multiple pregnancies, pregnancy complications, chronic illnesses, or a child with developmental disorders. We did not include review articles (scoping, narrative, meta-analysis), non-English articles, or conference proceedings and articles where full texts were unavailable. Two authors (AAT and WD) screened the searched articles using title and abstracts. Disagreements were solved by the third author (TAZ).

### Outcome

The primary outcome of this meta-analysis was child linear growth measured by length/height, height for age, or length for age evaluated at different time points in under-five children.

### Data extraction

Two independent authors (AAT and WD) extracted the data. Data extraction sheets containing relevant study characteristics and study outcomes were drafted into Covidence software. Disagreements were resolved by the third author (TAZ). Relevant information collected included author(s), publication year, study period, design, country, sample size, study outcomes, baseline maternal vitamin D status, initiation of supplementation, the dose of vitamin D, frequency of supplementation, duration of supplementation, maternal serum vitamin D concentration, child length/height, mean age, HFA/LFA, as well as the time of outcome evaluation in the experimental and comparison group.

### Quality assessment

The risk of bias for included clinical trials was judged by the Cochrane Collaboration Risk of Bias Tool [32], for reporting of sequence generation, allocation concealment, use of blinding of participants and personnel, loss to follow-up, and other biases. The methodological quality of the observational studies was assessed using the Newcastle Ottawa Scale [33], and the risk of bias in individual studies was rated as low, unclear risk, and high risk.

### Data analysis

Data analysis was dependent on the reporting system of the primary studies. Means of child length/height or length for age were pooled as weighted mean difference (WMD) in supplemented/high vitamin D and un-supplemented/low vitamin D groups. Some studies reported multiple treatment groups or reported deficient and insufficient vitamin D levels in observational studies. In both cases, the intervention group or deficient and insufficient vitamin D level sample size, mean length, and standard deviations were pooled [34].

Since there are studies that report child growth parameters at different time points, the WMD was calculated at different time points as well. We reported WMD with a 95% confidence interval (CI) using random effects, and the inverse variance method. Statistical heterogeneity was measured by I^2^ static, and we consider percentages of around I^2^ = 25%, I^2^ = 50%, and I^2^ = 75% as low, medium, and high heterogeneity, respectively [35]. Subgroup analysis was conducted to identify potential sources of clinical and methodological heterogeneity. This was performed on different variables, including study design, study area (continent), the dose of supplementation, trimester of pregnancy, subject recruitment time, and frequency of supplementation. To detect the robustness of the results, a sensitivity analysis was conducted by sequential elimination of each study from the pool. Potential publication bias was assessed using funnel plots, and where possible, Egger’s regression test was performed. The p-value ≤ 0.05 cut-point was used to declare statistical significance. The STATA software (Version 16, StataCorp, Texas, USA) was used for all analyses.

## Results

**Figure 1.**
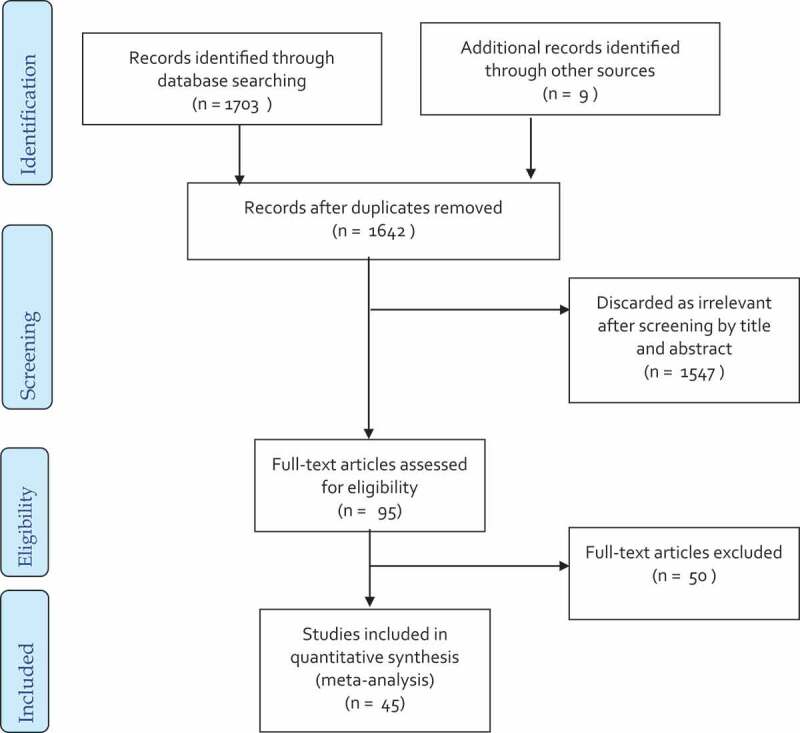
Flow chart describing the study selection process.

Overall, 1703 studies were identified through database searches, and nine additional articles were retrieved from the bibliographies of the included studies. Seventy duplicates were removed, and the remaining 1642 articles were screened by title and abstract, which resulted in the exclusion of 1547 irrelevant articles. Full-text screening was performed on 95 studies, and data for 45 studies were extracted for this meta-analysis. Figure 1 depicts the various exclusions and selection procedures.

The flow chart shows the stages of screening as well as numbers of articles excluded and included. The exclusion criteria included studies without a comparison group (18 articles), no outcome (13), giving wrong intervention (6), incomplete outcome (3), articles without full text or full text was unavailable (2), authors’ replies (2), intervention given with other nutrients (2), duplicate (1), studies investigating non-healthy children (1), review (1), and non-English language (1) articles were excluded after full-text screening. This sums up a total exclusion of 50 studies.

### Characteristics of included studies

A total of 45 studies and 66 reports were included in this meta-analysis. Twenty-five clinical trials and 20 observational studies with a total population of 44,992 (19,683 either intervention or high vitamin D group, and 25,309 control or low vitamin D group) have been analyzed. Included studies reported the outcome at different time points, including birth (40 studies 23 interventional [36–58] and 17 observational [59–75]), 1 month (three studies) [36,54,76], 3 months (five studies) [36,42,54,76,77], 6 months (four studies) [42,60,76,77], 9 months (four studies) [42,62,76,77], 1 year (five studies) [46,55,74,76,77] and five studies reported length for age [45,46,55,78,79]. The clinical trials were conducted between the years 1977 and 2015. The vast majority were randomized, and two-thirds were carried out in low- and middle-income countries such as Iran, India, and Bangladesh. Recruitment began as early as 10 weeks and finished as late as 32 weeks. Almost all clinical trials found that the baseline maternal vitamin D concentration was insignificant. A description of the included clinical trials is given in Table 1.

**Table 1.** Characteristics of included interventional studies.

	Author	Country	Year	Randomization	Blinding	Intervention initiation (Week)	Dose	Control	End	Measurement	Baseline vitamin D		Outcome
											Intervention	Control	
1	Abotorabi 2017	Iran	NE	Randomized	Non	22–26	50,000 IU/weekly+250 mg ca	400 IU/day+250 mg ca	Term	Birth	45 mmol/L	47.5 mmol/L	Anthropometry at birth
2	Brooke 1980	UK	1977–1979	Randomized	Double	28–32	1000 IU/day	Placebo	Term	Birth	20.2	20	Anthropometry at birth
3	Brooke 1981	UK	1977–1979	Randomized	Double	28–32	1000 IU/day	Placebo	Term	3 m, 6 m, 9 m, 12 m	20.2 nm/L	20 nm/L	Postnatal growth until 12 months
4	Brustad 2020	Denmark	2010	Randomized	Double	24	2800 IU/day	400 IU/day	1 weekpostpartum	B, 3y, 6 y	76.6 nm/L	76.4 nm/L	Anthropometric and bone outcomes
5	Charandabi 2015	Iran	2013–2014	Randomized	Triple	25–30	1000 IU/day	Placebo	60 days	Birth	NE	NE	Duration of pregnancy, type of delivery and infant anthropometric indicators
6	Cooper 2016	UK	2008–2014	Randomized	Double	10–17	1000 IU/day	Placebo	Delivery	Birth	46.7 nm/L	45.9 nm/L	Anthropometry and whole-body bone mineralization and composition in neonates
7	Diogenes 2015	Brazil	2009–2011	Randomized	Single	26	200 IU + 600 mg Ca/day	Placebo	Delivery	Birth, 5 w	59.5 nm/L	57.9 nm/L	Infant anthropometric and total body bone, maternal bone mineral density
8	Doria 2017	USA	2012–2013	Randomized	Double	24–28	3800 IU/daily	400 IU/daily	Up to 4–6 weeks post	Birth	31.5 ng/ml	32 ng/ml	Infant anthropometric, maternal and infant vitamin D status
9	Elmee 2017	Iran	2014–2016	Non-randomized	Not-blinded	14–24	50,000 IU/week	Placebo	Term	Birth	NE	<30 ng/ml	Anthropometry
10	Hajhashemi 2017	Iran	2015	Randomized	Non	14–18	4000 IU/day	30’ sun	10 weeks	Birth	15.95 ng/ml	15.09 ng/ml	Infant anthropometric and vitamin D level
11	Hashemipour 2014	Iran	2011–2012	Randomized	Single	24–26	Ca+D + 50,000 IU/day	Ca+D	8 weeks	Birth	<30 ng/ml	<30 ng/ml	Maternal weight gain, neonatal anthropometry
12	Hornsby 2017	USA	NE	Randomized	Double	10–18	4400 IU/day	400 IU/day	Delivery	Birth	19.2 ng/ml 20.3 ng/ml	23.5 ng/ml	Neonatal immunity
13	Hossian 2014	Pakistan	2010–2012	Randomized	NA	<20	And 4000 IU/daily	400 mg iron+600 mg Ca/daily	Delivery	Birth	4.74 ng/dl	5.31 ng/dl	Obstetric and neonatal outcomes
14	Kalra 2012	India		Partial Randomized	Single	12–24	Two doses of 3000 ng	Single 1500 ng	NE	Birth, 3, 6, 9 m	31.7 nm/L	32 nm/L	Alkaline phosphatase, neonatal serum Ca and anthropometry, maternal vitamin D at term
15	Karamali 2015	Iran	2014	Randomized	Double	20	5000 IU/fortnightly	Placebo	32 wks	Birth	16.99 ng/ml	17.1 ng/ml	Metabolic profiles and pregnancy outcomes
16	Litonjua 2016	USA	2009–2015	Randomized	Double	10–18	4000 IU+400 IU/daily	Placebo+400 IU/daily	NE	Birth	23.3 ng/ml	22.5 ng/ml	Asthma or recurrent wheeze, maternal vitamin D, child anthropometry
17	Mojibian 2015	Iran	2010–2012	Randomized	Not blinded	12	50,000 IU/fortnight	400 IU/daily	Delivery	Birth	14.46 ng/ml	15.31 ng/ml	Maternal complications and neonatal outcomes
18	Perumal 2015	Bangladesh	2010–2011	Randomized	Double	26–29	35,000 IU/week	Placebo	Delivery	Birth	NE	NE	Infant vitamin D at 6 months, anthropometrics at birth
19	Roth 2013	Bangladesh	2010–2012	Randomized	Double	26–29	35,000 IU/weekly	Placebo	Delivery	B, 4, 8, 16, 24, 36, 52 wks	38.5 nm/L	45.3 nm/L	Child anthropometrics until 5 years
20	Roth 2018	Bangladesh	2014–2015	Randomized	Double	17–24	4200–28,000 IU/week	Placebo	Delivery	B, 3, 6, 9, 12 m	28 nm/L	27.6 nm/L	Child anthropometrics until 1 year
21	Sabet 2012	Iran	2009–2010	Randomized	Double	27–28	100,000 IU/month	Placebo	Term	Birth	33.5 ng/ml	38.5 ng/ml	Vitamin D and iPTH, infant anthropometry
22	Sablok 2015	India	NE	Randomized	NE	14–20	60,000 IU (1), 120,000 IU (2), or 120,000 IU (4)	Placebo	Term	Birth	NE	NE	Cord blood vitamin D, neonatal anthropometry, SGA, preterm birth
23	Sahoo 2016	India	2012–2013	Randomized	Double	<20	60,000 u/4–8 weeks	Placebo	Delivery	B, 12–16 m	28.13 nm/L	28.5 nm/L	Bone mineral and body composition of offspring
24	Vaziri 2016	Iran	2014–2015	Randomized	Double	26–28	2000 IU/day	Placebo	Delivery	Birth, 4 w, 8 w	11.62 ng/ml	12.72 ng/ml	Anthropometrics and bone mass of mother-infant pairs
25	O’Callaghan 2022	Bangladesh	2014–2016	Randomized	Double blind	17–24	4200–28,000 IU/week	Placebo	Delivery	Birth, 4 years	28 nm/L	27.6 nm/L	Child anthropometrics and bone mineral density until 4 years

IU: international units, w: weeks, m: months, NE: not extractable, y: year

The design of observational studies was either cohort or cross-sectional. Maternal vitamin D levels were measured from 9 weeks after conception to full term. The definition of low and high vitamin D levels varied between studies. One study did not report the cut points, while another simply labeled vitamin D levels as adequate or inadequate. Three studies failed to provide length/height measurements at birth, although they were added subsequently, e.g. at 6 or 9 months post-birth. Table 2 lists the characteristics of the included observational studies.

**Table 2.** Characteristics of included observational studies.

	Study	Country	Design	Period	Recruitment time	Lower	Higher	Measurement time	Outcomes
1	Bogossian 2019	USA	Cohort	1992–1995	13–21 weeks	<20 ng/ml	≥20 ng/ml	Birth	Neonatal body composition including anthropometry at birth
2	Chi 2018	China	Cohort	2014–2015	28 weeks	<50 nm/L	≥50 nm/L	Birth	Neurodevelopment and anthropometry
3	Dalgard 2016	Denmark	Cohort	1997–2000,2007–2009	34–35 weeks	<25 nm/L	≥25 nm/L	14 days	Anthropometry
4	Gale 2008	UK	Cohort	1991–92	28–42 weeks	<50 nm/L	≥50 nm/L	Birth, 9 m, 9 y	Anthropometry, eczema, blood pressure, and cardiac structure
5	Jozwaik 2014	Poland	Cohort	NE	Third trimester	<30 ng/ml	≥30 ng/ml	Birth	Pregnancy outcome, health of newborns and mothers
6	Kilikaslan 2017	Turkey	C/S	2014	Term	<10 ng/ml	≥10 ng/ml	Birth	Birth parameters
7	Leffelaar 2010	Netherlands	Cohort	2003–2004	First ANC	<50 nm/L	≥50 nm/L	Birth, 1, 3, 6, 9, 12 m	Child anthropometry
8	Morales 2015	Spain	Cohort	2003–2008	13–15 weeks	<30 ng/ml	≥30 ng/ml	B, 1 y, 4 y	Anthropometry
9	Morley 2006	Australia	Cohort	2002–2003	28–32 weeks	<28 nm/L	≥28 nm/L	Birth	Newborn body composition
10	Ni 2021	China	C/S	2015–2016	9–13 weeks	<50 nm/L	≥50 nm/L	Birth	Neonatal outcomes
11	Ong 2016	Singapore	Cohort	NE	26–28 weeks	<50 nm/L	≥50 nm/L	Birth to 2 y, every 3 m	Birth outcomes, post-natal growth
12	Reichetzeder 2014	Germany	Cohort	2007–2008	Third trimester	<25 nm/L	≥25 nm/L	Birth	Birth outcomes
13	Sabour 2006	Iran	C/S	2004	Term	Inadequate	Adequate	Birth	Pregnancy outcome
14	Sarma 2018	India	Cohort	2012–2015	34 weeks	<30 ng/ml	≥30 ng/ml	Birth	Fetal skeletal size and growth
15	Shakeri 2018	Iran	C/S	2017	Third trimester	First and second	Third tercile	Birth	Weight gain, maternal biochemical parameters, and infants’ growth indices at birth
16	Song 2013	China	C/S	2010	Term	<25 nm/L	≥25 nm/L	Birth	Anthropometry
17	Viljakainen 2010	Finland	C/S	2007	First trimester	<42.6 ng/ml	≥42.6 ng/ml	Birth	Anthropometry and bone variables
18	Zhou 2014	China	Cohort	2011–2012	16–20 weeks	<30 ng/ml	≥30 ng/ml	Birth	Maternal, fetal, and neonatal outcome
19	Viljakainen 2011	Finland	Cohort	2007–2009	First trimester	<50 nm/L	≥50 nm/L	1 year	Anthropometry and bone turnover markers
20	Eckhardt 2014	USA	Cohort	1959–1965	≤26 weeks	<30 nmol/L	≥30 nmol/L	Birth, 4 m, 4 year	Child anthropometry

ANC: antenatal care, c/s: cross-sectional, m: month, NE: not extractable, y: year

### Meta-analysis

**Figure 2.**
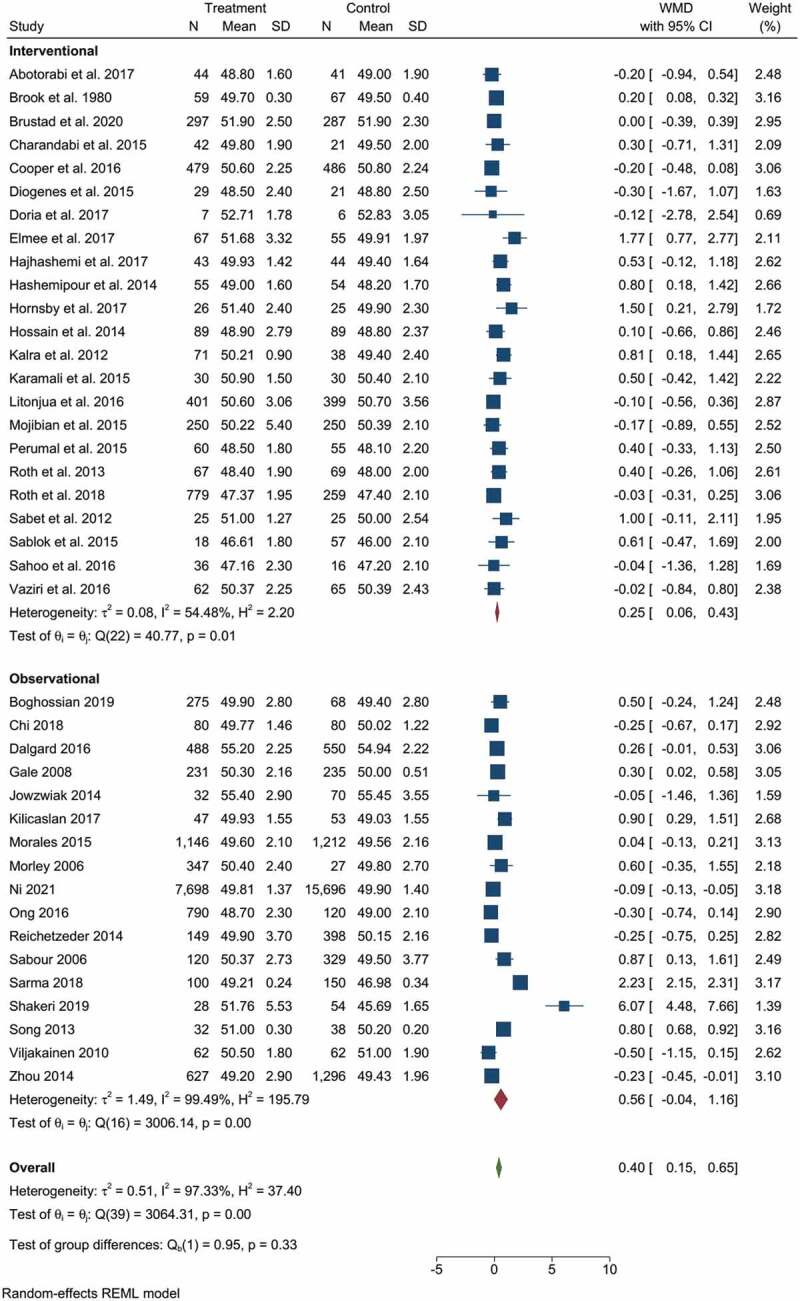
The forest plot shows the effect of vitamin D supplementation/high vs low on birth length. The graph indicates the overall important effect of the vitamin to promote linear growth. The first subgroup represents clinical trials, and the second includes observational studies.

The pooled results from clinical trials and observational studies indicated the beneficial effect of vitamin D supplementation/higher vitamin D status during pregnancy for the linear growth of children. The pooled effect size from 23 clinical trials and 17 observational studies had a WMD of 0.4 cm birth length with a (95% CI: 0.15–0.65), and I^2^ statistics of 97.33%. Children whose mothers were supplemented with various doses of vitamin D during pregnancy, or had sufficient vitamin D, showed a significant increase in birth length (p-value < 0.001), indicated in Figure 2.

Subgroup analysis showed that prenatal vitamin D supplementation had a significant effect on childbirth length. Figure 2 shows that mothers who took vitamin D supplements had longer children with WMD = 0.25 cm (95% CI: 0.06–0.43 cm) and I^2^ static = 54.48%.

According to the findings of observational studies, there is no statistically significant difference in birth length between mothers with high and low levels of vitamin D. WMD = 0.56 cm (95% CI: −0.04 cm to 1.16 cm) (Figure 2). The pooled analysis also indicated significant heterogeneity, with I^2^ = 99.49%. Neither subgroup analysis based on study area (developing vs developed), design (cohort vs cross-sectional), or vitamin D category (the authors’ criteria for classifying high and low) produced significant results or significantly reduced heterogeneity.

**Figure 3.**
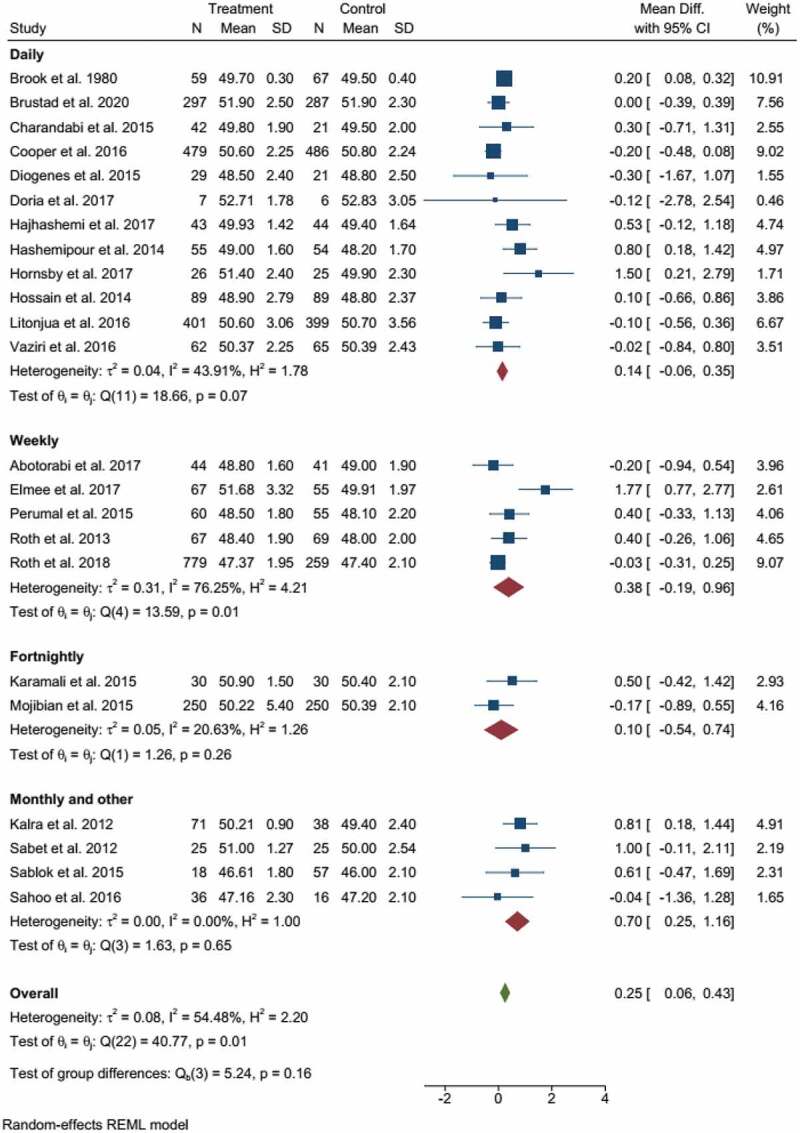
Forest plot of subgroup analysis based on the frequency of supplementation.

Subgroup analysis based on the frequency of supplementation indicated the significant effect of intermittent supplementation (monthly or less frequent) on childbirth length. Mothers who supplemented monthly or less frequently had a 0.7 cm longer child with (95% CI: 0.25−1.16 cm) of I^2^ = 0.00%, given in Figure 3. Subgroup analysis with the dose of supplementation also revealed that supplementation with a dose of >2000 IU contributed to child length at birth, WMD = 0.35 cm (95% CI: 0.11−0.58 cm), and I^2^ = 49.82%; given in the supplementary file, SFigure 1.

Overall, higher maternal vitamin D or Vitamin D supplementation from 20 weeks of gestation had a significant positive effect on birth length. Subgroup analysis from the clinical trials indicated a significant effect of vitamin D supplementation either below or above 20 weeks of gestation. Clinical trials that supplemented vitamin D less than 20 weeks of gestation had a greater effect size (WMD, 0.38 cm vs 0.17 cm) (see the supplementary file, SFigure 2).

As previously stated, some studies report child length after birth. Table 3 summarizes these findings. As can be seen, maternal vitamin D had a significant effect on child length at 3 months. Aside from this overall effect, observational studies at 6 months and both interventional and observational studies separately at 9 months reported a positive influence of higher maternal vitamin D levels (see Table 3). In contrast to what we saw in this meta-analysis, observational studies revealed a negative effect of higher maternal vitamin D on child growth at 12 months of age (WMD = −0.05 cm (95% CI: −0.06 cm to −0.04 cm), I^2^ = 0.00%) (Table 3). The forest plots for these outcomes are included in the supplementary file (SFigure 5−SFigure 10).

**Table 3.** The role of maternal vitamin D on child linear growth beyond birth disaggregated by study design.

No.	Age	Design	Number of studies	Mean difference, IV random, 95% CI	I^2^ (p-value)
1	1 month	Interventional	2	0.2(−0.74,0.34)	0.001(0.58)
		Observational	1	*0.62(0.1, 0.63)*	-
		Total	3	0.19(−0.43, 0.82)	74.3(0.001)
2	3 months	Interventional	4	0.51(−0.18, 1.21)	67.3(0.02)
		Observational	1	*0.41(0.40, 0.42)*	-
		Total	5	*0.50(0.03, 0.97)*	71.39(0.02)
3	6 months	Interventional	2	1.33(−0.30, 2.96)	86.75(0.01)
		Observational	2	*0.2(0.19, 0.21)*	0.00(0.58)
		Total	4	0.78(−0.08, 1.65)	87.85(0.001)
4	9 months	Interventional	2	*1.48(0.13, 2.82)*	79.77(0.03)
		Observational	2	*0.1(0.09, 0.11)*	0.00(1.0)
		Total	4	0.73(−0.09, 1.65)	92.83(0.001)
5	12 months	Interventional	3	0.75(−0.35, 1.92)	88.49(0.001)
		Observational	2	−0.05(−0.06, −0.04)	0.00(0.42)
		Total	5	0.37(−0.47, 1.17)	93.6(0.001)
6	LFA	Interventional	4	0.01(−0.23, 0.25)	60.65(0.10)
		Observational	1	*0.18(0.07, 0.29)*	-
		Total	5	0.06(−0.13, 0.25)	70.01(0.01)

CI: confidence interval, LFA: length for age, IV: inverse variance, LFA: length for age

## Publication bias and small study effects

**Figure 4.**
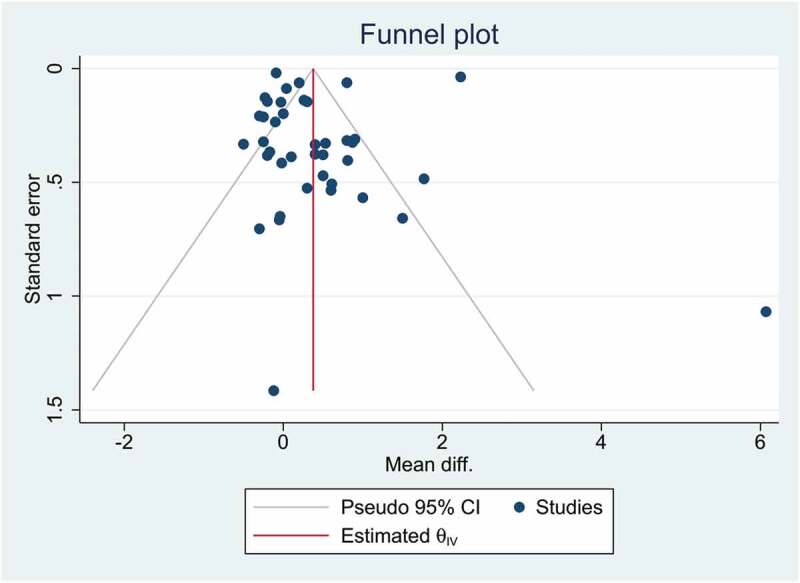
Funnel plot.

Figure 4 shows a funnel plot for visual inspection of publication bias. In addition, the Eggers regression test was used to detect publication bias and small-study effects. According to the findings, there was no publication bias and small-study effects (p-value = 0.2414).

## Discussion

Results from the pooled analysis of clinical trials and observational studies indicated beneficial effects of vitamin D supplementation in pregnancy on the linear growth of children. Children whose mothers were supplemented with various doses of vitamin D during pregnancy or who already had sufficient vitamin D showed a significant increase in birth length (p-value < 0.001). Previous systematic reviews highlighted the beneficial effects of vitamin D supplementation or higher levels of vitamin D during pregnancy on preeclampsia, preterm birth, small for gestational age [80], birth weight and length, gestational diabetes [81], cesarean section [82], enhanced cognitive development, and lower risk of attention deficit hyperactivity disorder and autism-related traits later in life [83]. Other studies [26,84] have questioned the role of prenatal vitamin D supplementation in the risk of cesarean section, gestational diabetes, stillbirth, neonatal death, and child respiratory health.

Overall, maternal vitamin D supplementation appears to increase child length at birth. Although there are no previous comprehensive meta-analyses to compare with the current findings, a few studies evaluated the growth-promoting effect of vitamin D as a secondary outcome. Bi et al. reported significantly greater height at 3 months, 9 months, and 12 months, but not at 6 months [82]. A meta-analysis from four clinical trials indicated that the LFA z-score was higher in infants at 1 year in the vitamin-D-supplemented group compared with the control [85]. Vitamin D supplementation at a higher dose and on an intermittent basis was found to be more beneficial than a lower dose (2000 IU) and daily or weekly supplement. Daily vitamin D is often inadequate to treat vitamin D deficiency due to compliance [83]. Despite the scarcity of studies on pregnant women, various studies have stressed the importance of large single doses of vitamin D in different populations. In their review that investigated the effects of single, large doses of vitamin D on serum concentrations and other health outcomes, Kearns et al. [86] came to the conclusion that a single vitamin D3 dose ≥300,000 IU was most effective at improving vitamin D status for up to 3 months in adults. In line with this finding, Boonen et al. [87] reported cholecalciferol 100,000 IU was a safe, effective, and simple way to increase serum vitamin D for up to 2 months in the elderly. Other studies have found that daily, weekly, and monthly administrations of the daily equivalent of 1000 IU of vitamin D3 provide equal efficacy and safety profiles, with intermittent supplementation still being preferred [29,88].

The effect of maternal vitamin D on child growth was significant when initiated or measured at >20 weeks of gestation. Similar findings were reported in previous meta-analyses on different outcomes [82]. Vitamin D supplementation increased birth weight only in the group with therapy initiated late (≥20 weeks’ gestation). Evidence that higher maternal vitamin D levels in later trimesters were associated with better outcomes suggests the need to monitor maternal vitamin D beyond the first trimester. Higher maternal vitamin D in the first trimester is not necessarily an indication of subsequent status during pregnancy. This was shown in clinical trials where the initiation of supplementation of vitamin D at less than 20 weeks of gestation had a greater effect size (WMD, 0.38 cm vs 0.17 cm) (supplementary file, SFigure 3). This underscores the importance of continuous vitamin D monitoring considering the plasma half-life.

This study adds to our existing knowledge of maternal vitamin D and its role in child development. Our review includes both interventional and observational studies. The risk of bias and methodological quality of included studies are summarized in supplementary files (STable 1 and STable 2). The focus was on linear growth as an outcome to provide us with a comprehensive understanding of this issue. This study also demonstrated the role of various factors such as supplementation dose, time of initiation, frequency of supplementation, and trimester. The findings suggest that the focus should be on higher vitamin D doses, earlier initiation, and sustained adequate levels, as well as less frequent supplementation.

In light of all of this, the following limitations should be considered when interpreting the results. First, there is significant heterogeneity. The included studies differed in many ways, including the populations studied, ethnicity, geographic factors, maternal vitamin D dose and cutoff points, clinical settings, the timing of intervention and/or measurement, and baseline maternal factors such as socioeconomic indicators.

Second, even though the objective of the meta-analysis was to assess linear growth in under-five children, there were a limited number of reports after birth. Few studies reported length at 1 month (3), 3 months (5), 6 months (4), and 1 year (5), and five studies reported length for age. The effect of maternal vitamin D on child growth beyond 12 months of age was not incorporated due to the lack of available studies. Despite our initial concept of the source of vitamin D in children, during the first 6 to 8 weeks of postnatal life, the vitamin D status of infants is mainly dependent on placental transfer in utero [21]. As previously noted, stores are depleted by approximately 8 weeks of age [22], after which time the infant’s vitamin D is dependent on diet, sunlight, and supplementation. This temporal relationship was not established in the data due to lack of available studies.

Third, another critical issue in this meta-analysis is adherence. This paper signified the importance of monthly or less frequent supplementation rather than daily or weekly. We hypothesized that this might be due to adherence. However, this was not confirmed here due to limited information regarding adherence. A prospective cohort study hypothesized that a 5000 IU daily supplement is superior to the 200,000 IU stat supplement and recommended that randomized control trials be conducted in order to confirm this hypothesis [89]. A controlled trial reported that a single 5 mg dose of vitamin D given orally during the seventh month of pregnancy provided effective prophylaxis for vitamin D deficiency over 1000 IU daily supplement [90]. A review also suggested that prenatal vitamin D supplementation with a higher dose could be reformulated due to several factors, the major one being adherence [27]. The evidence on adherence is mixed. Daily supplementation has been shown to have poor adherence [91]. In a 2001 study of protease inhibitor regimen adherence among HIV patients, for example, true adherence via electronic monitors was 63%, while pill count indicated 83% adherence [92]. These findings support evidence of the poor adherence but also call the verification methods into question.

## Conclusion

The evidence suggests that prenatal vitamin D supplementation in higher doses (>2000 IU), low frequency (monthly or less frequently), and later gestation (>20 weeks) is positively associated with higher child length/height. There is, however, a need for further evidence from clinical trials, not only comparing different doses and frequencies but also investigating adherence. In summary, the evidence to date suggests that consistent and adequate levels of vitamin D during pregnancy are critical for children’s growth.

## Supplementary Material

Supplemental Material

## Paper context

Prenatal intake of vitamin D has positive effects on birth outcomes. Vitamin D supplementation during pregnancy is associated with higher birth length and the effects are more evident at higher doses, low frequency, and in later stages of pregnancy. Consistent and adequate vitamin D levels are crucial in pregnancy. Future clinical trials are needed to evaluate frequency, dose, and adherence.
