# Supplementary material for: Maternal vitamin D and growth of under-five children: a systematic review and meta-analysis of observational and interventional studies
Source: Glob Health Action. 2022 Aug 31;15(1):2102712. doi: 10.1080/16549716.2022.2102712 (PMC9448399; doi:10.1080/16549716.2022.2102712)
Supplement: Supplemental Material [file ZGHA_A_2102712_SM9554.docx]

Supplementary materials

Table of Contents

[Risk of bias for clinical trials 2](#_Toc103101352)

[Quality assessment of observational studies using Newcastle Ottawa Scale 3](#_Toc103101353)

[Sub group analysis 4](#_Toc103101354)

[Results of growth beyond birth 8](#_Toc103101355)

# STable 1: Risk of bias for clinical trials

|  | Author | Sequence generation | Allocation concealment | Blinding of participants and personnel | Blinding of outcome assessment | Incomplete outcome data | Selective outcome data | Other sources of bias |
| --- | --- | --- | --- | --- | --- | --- | --- | --- |
| 1 | Abotorabi 2017 | Low | High | Low | Unclear | Low | Low | Unclear |
| 2 | Brooke 1980 | Low | Low | Low | Low | High | Low | Low |
| 3 | Brooke 1981 | Low | Low | Low | Low | High | Low | Low |
| 4 | Brustad 2020 | Low | Low | Low | Low | Low | Low | Unclear |
| 5 | Charandabi 2015 | Unclear | low | Low | Low | Low | Low | High |
| 6 | cooper 2016 | Low | Low | Low | Low | Low | Low | Unclear |
| 7 | Diogenes 2015 | Unclear | High | Low | Low | Low | High | Low |
| 8 | Doria 2017 | Low | Low | Low | Low | Low | Low | High |
| 9 | Elmee 2017 | High | High | Unclear | Unclear | Low | Low | High |
| 10 | Hajhashemi 2017 | High | High | Unclear | Unclear | Low | Low | High |
| 11 | Hashemipour 2014 | Low | Unclear | High | Unclear | Low | Low | Unclear |
| 12 | Hornsby 2017 | Low | Low | Low | Low | Low | Low | Low |
| 13 | Hossian 2014 | High | Unclear | High | Unclear | Low | Low | High |
| 14 | Kalra 2012 | Low | Low | Low | Low | Low | Low | Low |
| 15 | Karamali 2015 | Low | Low | Low | Low | Low | Low | Low |
| 16 | Litonjua 2016 | Low | Low | Low | Low | Low | Low | Low |
| 17 | Mojibian 2015 | Low | Low | High | Low | Low | Low | Unclear |
| 18 | Perumal 2015 | Low | Low | Low | Low | Low | Low | Low |
| 19 | Roth 2013 | Low | Low | Low | Low | Low | Low | Low |
| 20 | Roth 2018 | Low | Low | Low | Low | Low | Low | Low |
| 21 | Sabet 2012 | Unclear | Unclear | Unclear | Unclear | Low | Low | Low |
| 22 | Sablok 2015 | Low | High | Unclear | Unclear | Low | Low | Unclear |
| 23 | Sahoo 2016 | Low | Low | Low | Low | High | Low | Unclear |
| 24 | Vaziri 2016 | Low | Low | Low | Low | Low | Low | Low |
| 25 | O’Callaghan 2022 | Low | Low | Low | Low | Low | Low | Low |

# STable 2: Quality assessment of observational studies using Newcastle Ottawa Scale

|  | Study | Selection | | |  | Comparability | | | |  | | Assessment of outcome | | | | | Total score |
| --- | --- | --- | --- | --- | --- | --- | --- | --- | --- | --- | --- | --- | --- | --- | --- | --- | --- |
|  |  | a | b | c | | | d |  | e | | f | |  | g | h | i |  |
| 1 | Bogossian 2019 | 1 | 1 | 1 | | | 0 |  | 1 | | 0 | |  | 1 | 1 | 0 | 6 |
| 2 | Chi 2018 | 1 | 1 | 1 | | | 0 |  | 1 | | 1 | |  | 1 | 0 | 1 | 7 |
| 3 | Dalgard 2016 | 1 | 1 | 1 | | | 1 |  | 1 | | 1 | |  | 1 | 0 | 1 | 8 |
| 4 | Eckhardt 2015 | 1 | 1 | 1 | | | 1 |  | 1 | | 1 | |  | 1 | 1 | 1 | 9 |
| 5 | Gale 2008 | 1 | 1 | 1 | | | 1 |  | 1 | | 1 | |  | 1 | 1 | 0 | 8 |
| 6 | Jozwaik 2014 | 1 | 1 | 1 | | | 1 |  | 1 | | 1 | |  | 1 | 0 | 0 | 7 |
| 7 | Kilikaslan 2017 | 1 | 1 | 1 | | | 1 |  | 1 | | 1 | |  | 1 | 0 | 0 | 7 |
| 8 | Leffelaar 2010 | 1 | 1 | 1 | | | 1 |  | 1 | | 1 | |  | 1 | 1 | 0 | 8 |
| 9 | Morales 2015 | 1 | 1 | 1 | | | 1 |  | 1 | | 1 | |  | 1 | 1 | 1 | 9 |
| 10 | Morley 2006 | 1 | 1 | 1 | | | 1 |  | 1 | | 1 | |  | 1 | 1 | 0 | 8 |
| 11 | Ni 2021 | 0 | 0 | 1 | | | 1 |  | 1 | | 1 | |  | 1 | 1 | 1 | 7 |
| 12 | Ong 2016 | 1 | 1 | 1 | | | 1 |  | 1 | | 1 | |  | 1 | 0 | 0 | 7 |
| 13 | Reichetzeder 2014 | 1 | 1 | 1 | | | 1 |  | 1 | | 1 | |  | 1 | 0 | 1 | 8 |
| 14 | Sabour 2006 | 1 | 1 | 0 | | | 1 |  | 0 | | 0 | |  | 1 | 0 | 1 | 5 |
| 15 | Sarma 2018 | 1 | 1 | 1 | | | 1 |  | 0 | | 0 | |  | 1 | 0 | 1 | 6 |
| 16 | Shakeri 2018 | 1 | 1 | 1 | | | 1 |  | 0 | | 0 | |  | 1 | 1 | 1 | 7 |
| 17 | Song 2013 | 0 | 1 | 1 | | | 1 |  | 0 | | 0 | |  | 1 | 0 | 1 | 5 |
| 18 | Viljakainen 2010 | 1 | 1 | 0 | | | 1 |  | 1 | | 1 | |  | 1 | 1 | 1 | 8 |
| 19 | Zhou 2014 | 1 | 1 | 1 | | | 1 |  | 0 | | 0 | |  | 1 | 1 | 0 | 6 |
| 20 | Viljakainen 2011 | 1 | 1 | 0 | | | 1 |  | 1 | | 1 | |  | 1 | 1 | 1 | 8 |

a. Representativeness of the exposed cohort;

b. Selection of the non-exposed cohort;

c. Ascertainment of exposure;

d. Demonstration that outcome of interest was not present at start of study;

e. Comparability of cohorts on the basis of the design or analysis (adjusted for maternal pre-pregnancy BMI/weight);

f. Comparability of cohorts on the basis of the design or analysis (adjusted for others);

g. Assessment of outcome;

h. Was follow-up long enough for outcomes to occur (maternal 25(OH)D level was measured before 20 weeks);

i. Adequacy of follow-up of cohort (follow up rate ≥80%).

# Sub group analysis


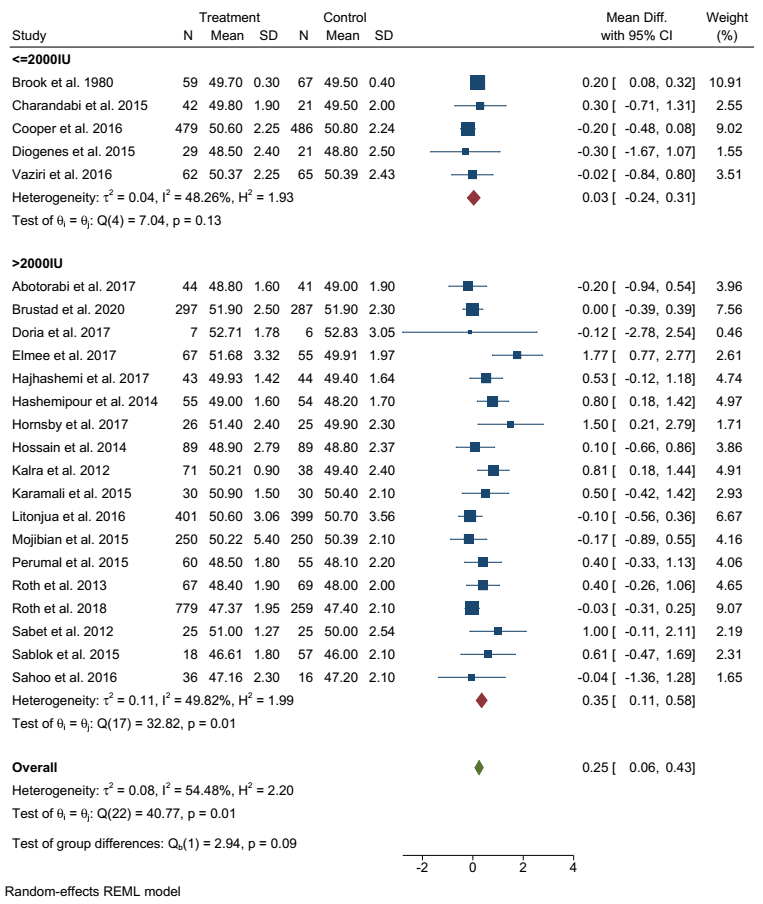


SFigure 1: Subgroup analysis based on dose of supplementation


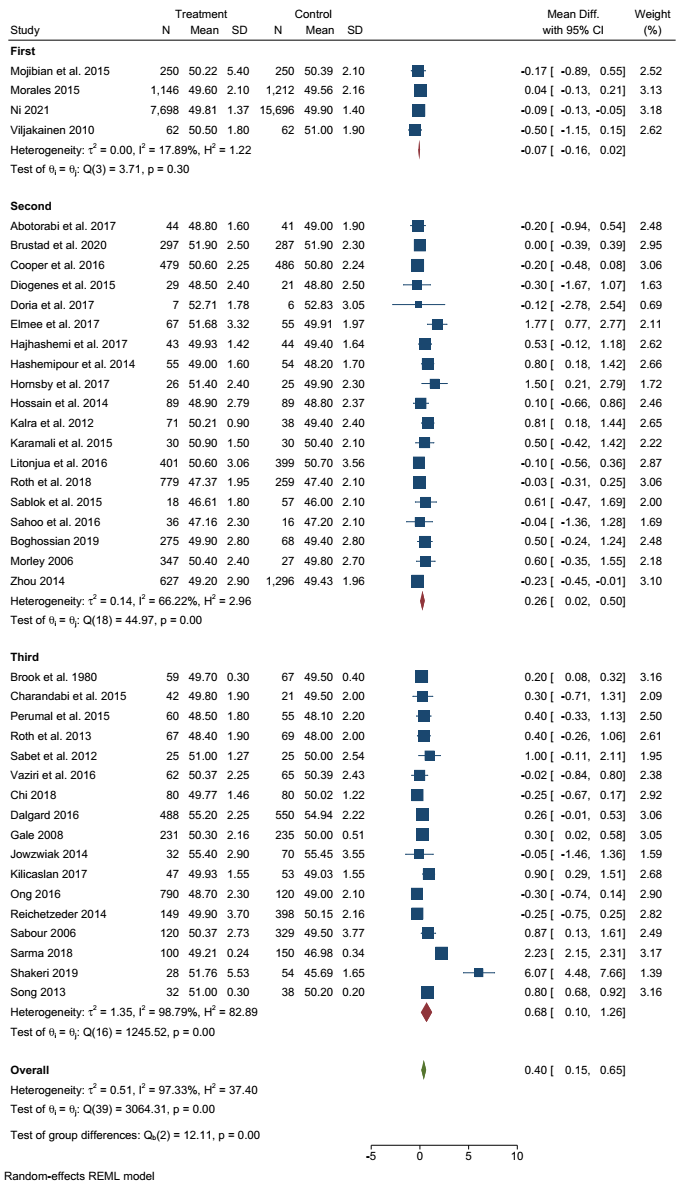


SFigure 2: Subgroup analysis based on trimester


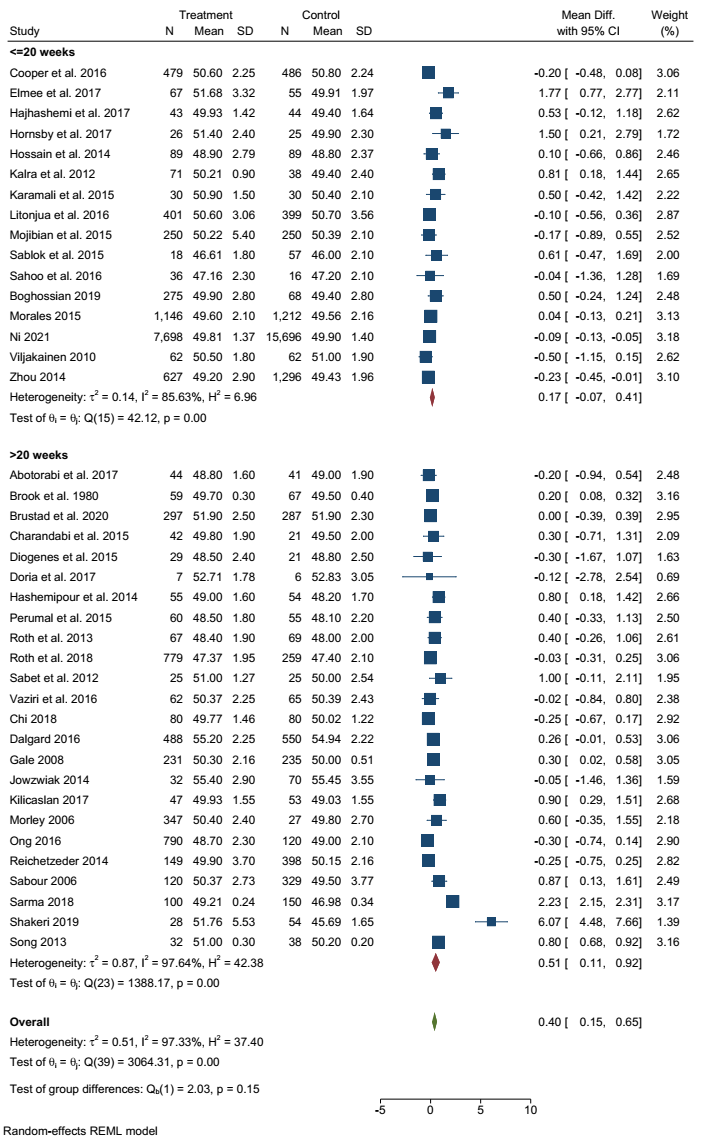


SFigure 3: Based on follow up/recruitment time of both observational and interventional studies


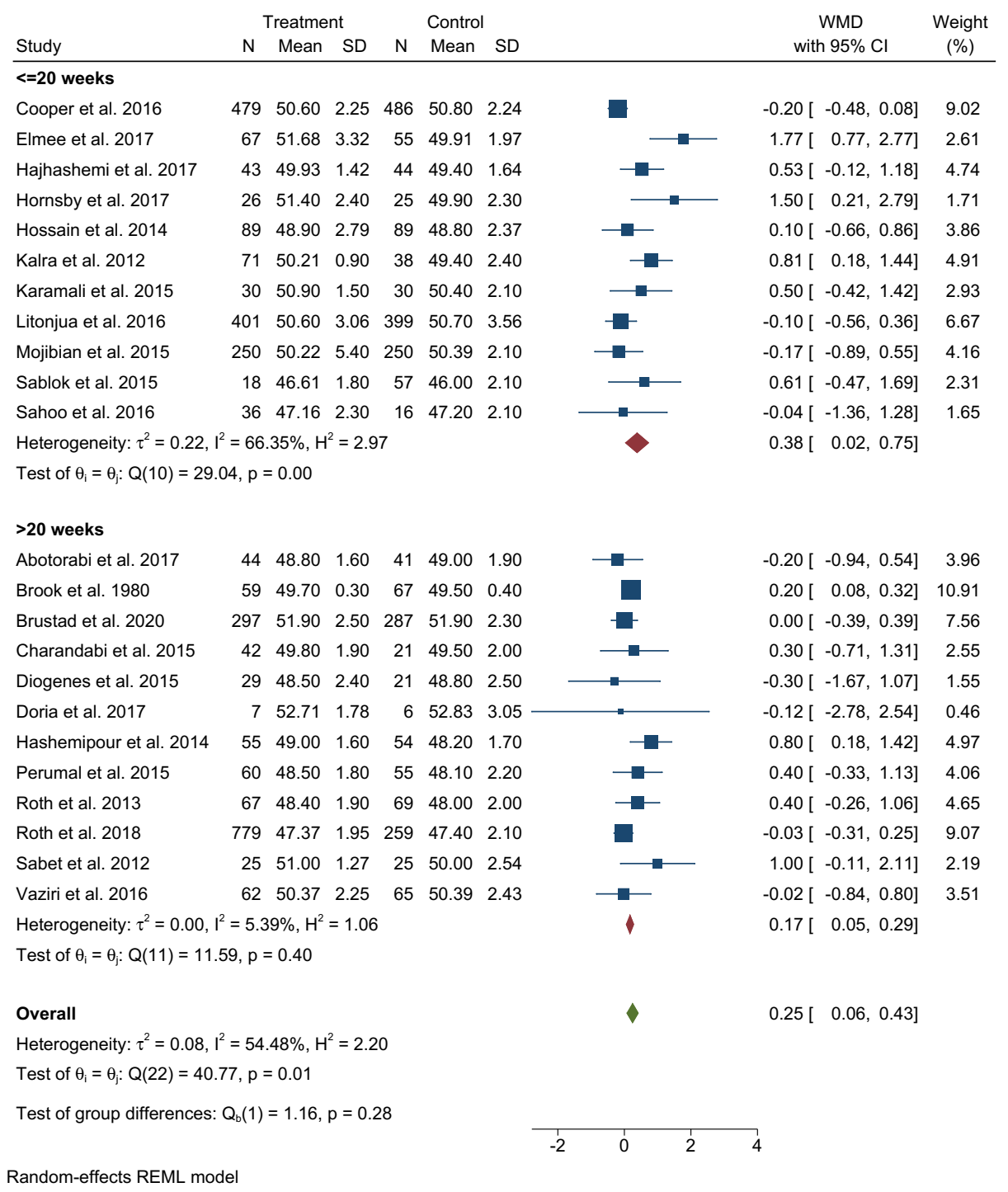


SFigure 4: Subgroup analysis of RCTs by subject recruitment time

# Results of growth beyond birth


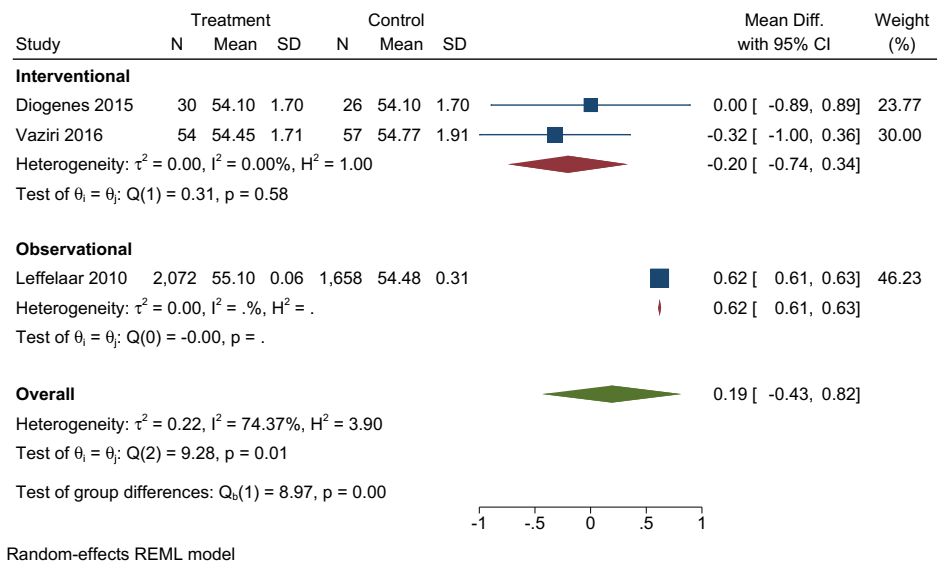


SFigure 5: Maternal vitamin D and child length at the first month


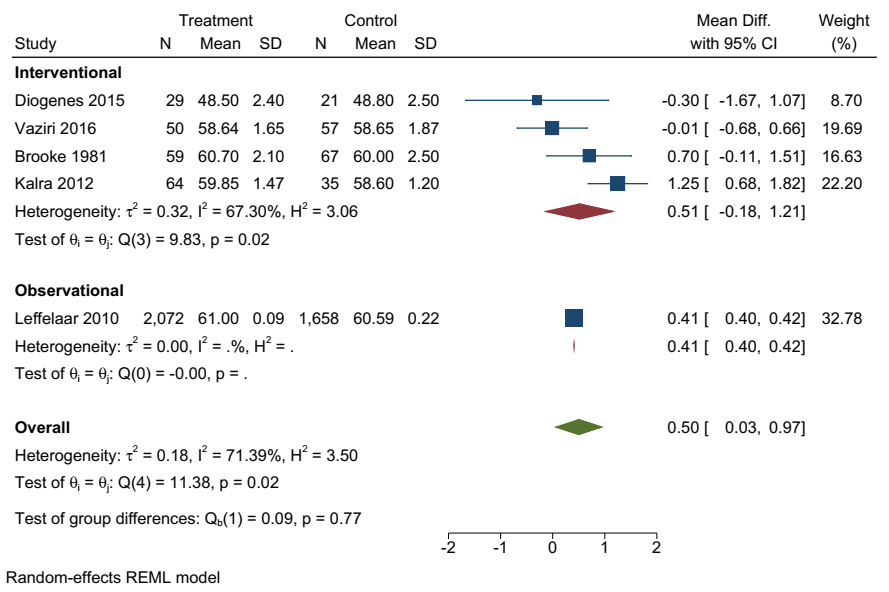


SFigure 6: Maternal vitamin D and child length at the three months


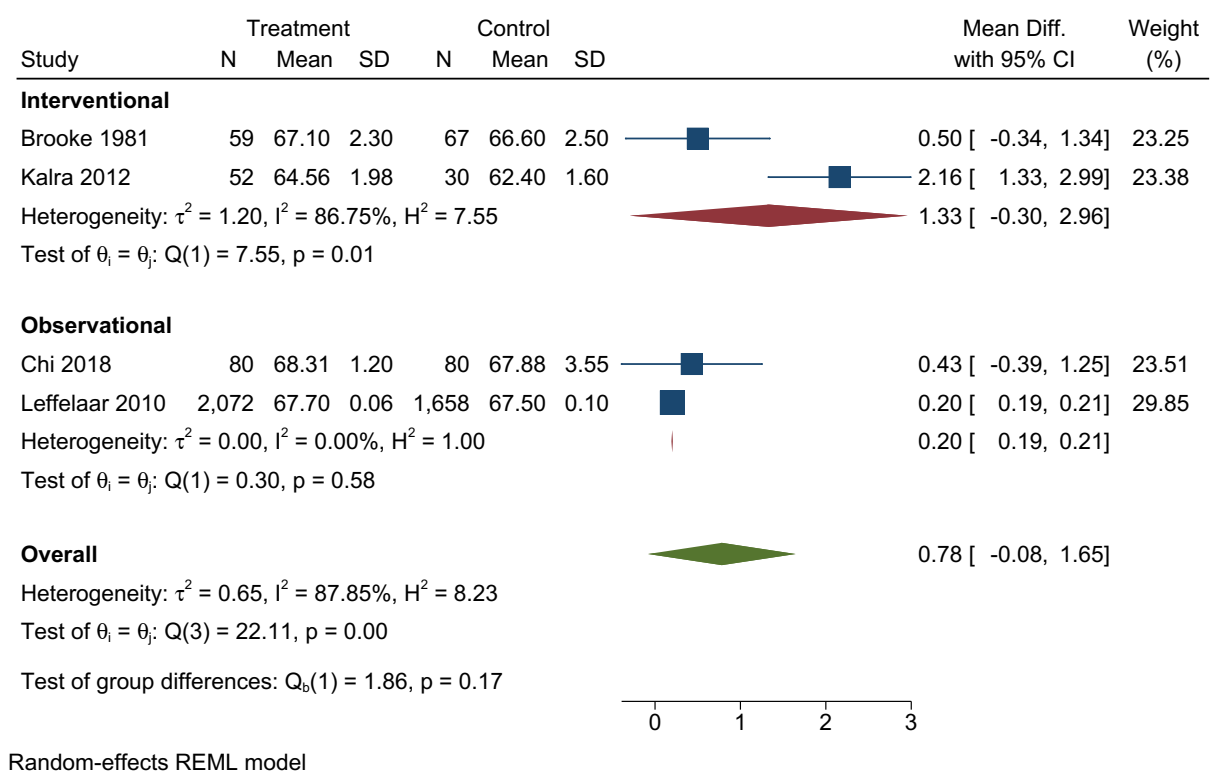


SFigure 7: Maternal vitamin D and child length at the six months


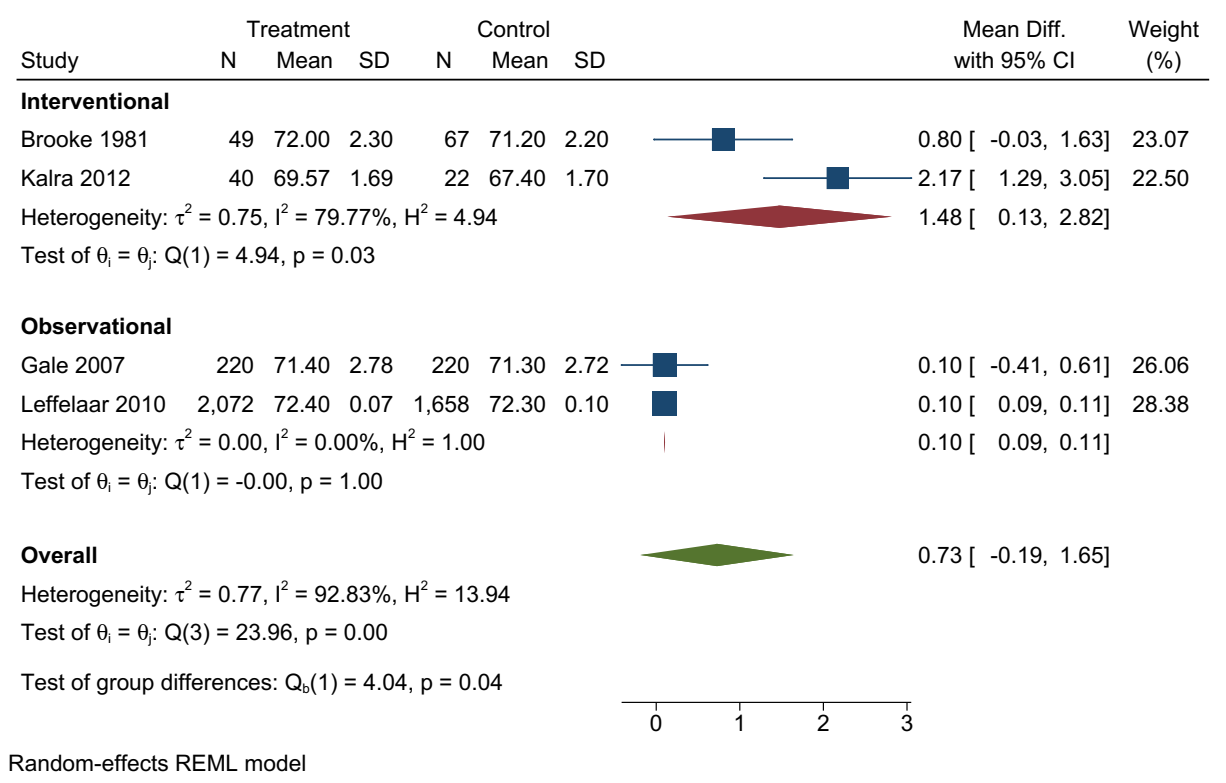


SFigure 8: Maternal vitamin D and child length at the nine months


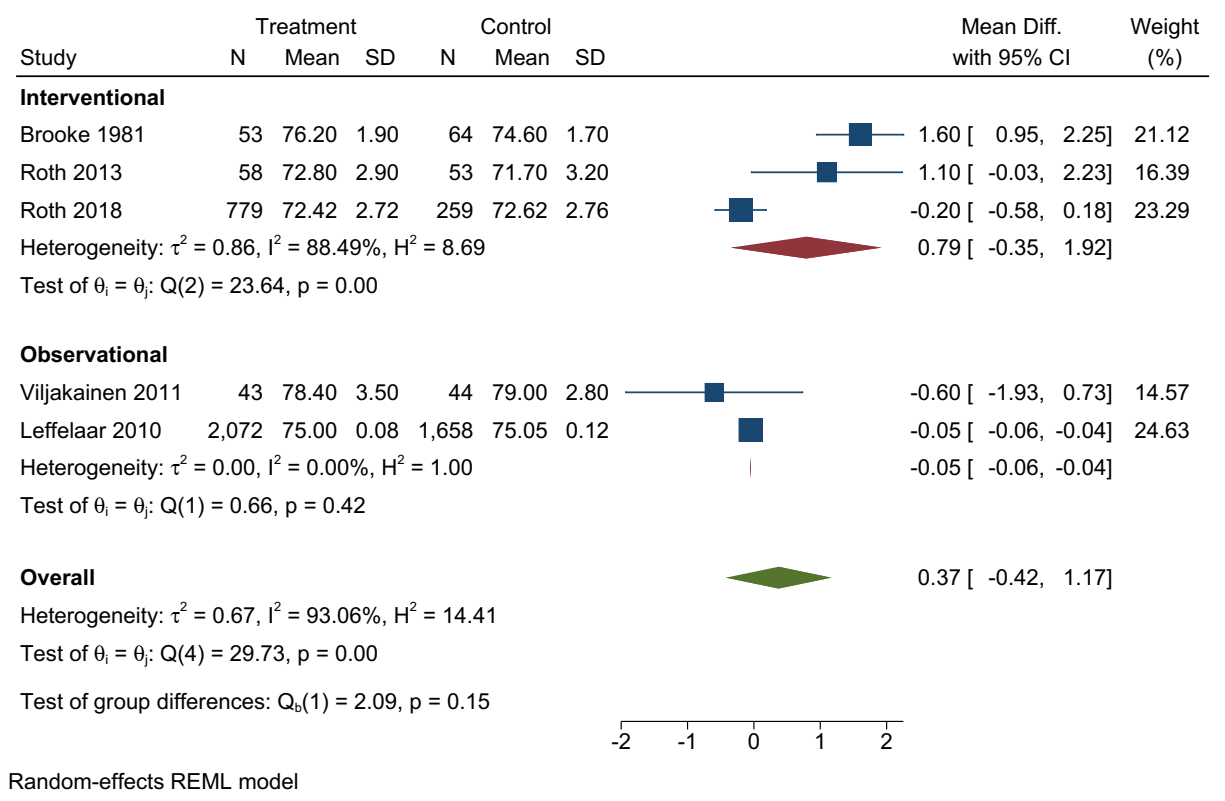


SFigure 9: Maternal vitamin D and child length at the twelve months


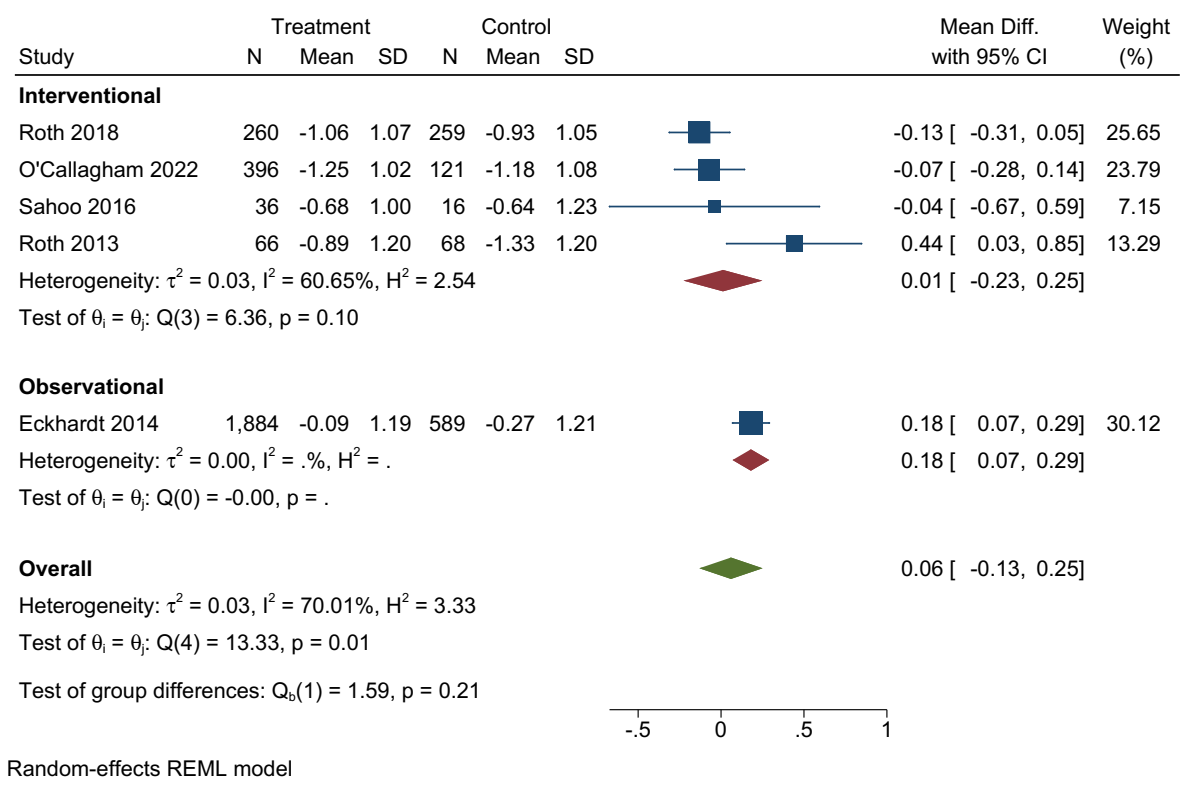


SFigure 10: Maternal vitamin D and child length for age
